# Auxin regulates adventitious root formation in tomato cuttings

**DOI:** 10.1186/s12870-019-2002-9

**Published:** 2019-10-21

**Authors:** Ling Guan, Reuben Tayengwa, Zongming (Max) Cheng, Wendy Ann Peer, Angus S. Murphy, Mizhen Zhao

**Affiliations:** 1 Institute of Pomology, Jiangsu Academy of Agricultural Sciences / Jiangsu Key Laboratory for Horticultural Crop Genetic improvement, Nanjing, 210014 China; 2 Department of Plant Science and Landscape Architecture, University of Maryland, College Park, MD USA; 3 College of Horticulture, Nanjing Agricultural University, Nanjing, 210095 China; 4 Department of Plant Sciences, University of Tennessee, Knoxville, TN USA; 5 Department of Environmental Science and Technology, University of Maryland, College Park, MD USA; 6 Agriculture Biotechnology Center, University of Maryland, College Park, MD USA

**Keywords:** Adventitious root, Auxin, Cutting, Root development, Pericycle, Tomato, Propagation

## Abstract

**Background:**

Adventitious root (AR) formation is a critical developmental process in cutting propagation for the horticultural industry. While auxin has been shown to regulate this process, the exact mechanism and details preceding AR formation remain unclear. Even though AR and lateral root (LR) formation share common developmental processes, there are exist some differences that need to be closely examined at the cytological level. Tomato stem cuttings, which readily form adventitious roots, represent the perfect system to study the influence of auxin on AR formation and to compare AR and LR organogenesis.

**Results:**

Here we show the progression by which AR form from founder cells in the basal pericycle cell layers in tomato stem cuttings. The first disordered clumps of cells assumed a dome shape that later differentiated into functional AR cell layers. Further growth resulted in emergence of mature AR through the epidermis following programmed cell death of epidermal cells. Auxin and ethylene levels increased in the basal stem cutting within 1 h. Tomato lines expressing the auxin response element DR5pro:YFP showed an increase in auxin distribution during the AR initiation phase, and was mainly concentrated in the meristematic cells of the developing AR. Treatment of stem cuttings with auxin, increased the number of AR primordia and the length of AR, while stem cuttings treated with the pre-emergent herbicide/auxin transport inhibitor N-1-naphthylphthalamic acid (NPA) occasionally developed thick, agravitropic AR. Hormone profile analyses showed that auxin positively regulated AR formation, whereas perturbations to zeatin, salicylic acid, and abscisic acid homeostasis suggested minor roles during tomato stem rooting. The gene expression of specific auxin transporters increased during specific developmental phases of AR formation.

**Conclusion:**

These data show that AR formation in tomato stems is a complex process. Upon perception of a wounding stimulus, expression of auxin transporter genes and accumulation of auxin at founder cell initiation sites in pericycle cell layers and later in the meristematic cells of the AR primordia were observed. A clear understanding and documentation of these events in tomato is critical to resolve AR formation in recalcitrant species like hardwoods and improve stem cutting propagation efficiency and effectiveness.

**Electronic supplementary material:**

The online version of this article (10.1186/s12870-019-2002-9) contains supplementary material, which is available to authorized users.

## Background

The root has multiple functions during plant growth and development including water and nutrient absorption. Depending on when and from which tissue they originate, roots can be defined as embryonic or post-embryonic [1]. Adventitious roots (AR) are post-embryonic roots which form at multiple sites in diverse organs including leaves, the root-shoot junction, stems in contact with the soil surface, and at the base of stem cuttings [2]. Adventitious roots can also form in response to abiotic stresses such as waterlogging or when embryonic roots are dysfunctional [3, 4].

Adventitious root formation is generally divided into three developmental phases: induction, initiation and extension [5]. During the induction phase, the primordium initial cells are established via de-differentiation of pericycle cells or cambium cells (this depends on the species and the age of the stem cutting) followed by cell division [6, 7]. In the initiation phase the meristematic cells of the primordia divide and differentiate into root cell layers [6]: epidermis, cortex, endodermis, vasculature, meristem and root cap [6, 8]. Finally, during the extension phase, AR primordia grow through the stem’s cell layers and emerge from the epidermis [6, 9]. While auxin (indole-3-acetic acid, IAA) has been shown to regulate AR formation during these three phases and almost every developmental step [8, 10], the detailed cytology and mechanism of AR formation in species other than *Arabidopsis thaliana* have not been well-described. Tomato stem cuttings readily form adventitious roots, which makes them an ideal system to study AR formation in detail.

Cell-to-cell auxin transport is mediated by a network of auxin influx and efflux carriers that are regulated at the transcriptional and post-translational levels [11]. There are three classes of auxin carriers and transporters at the plasma membrane. Two major classes exhibit auxin-efflux activity: the plant-specific PIN family of efflux carriers and the ATP-binding cassette (ABC) superfamily of transporters, predominantly the B-type (ABCB/multidrug resistance [MDR]/phosphoglycoprotein [PGP]). The AUXIN1/LIKE-AUX1 (AUX/LAX) gene family encodes auxin influx symporters. PIN proteins play an important role in polar auxin transport (PAT) due to their asymmetric subcellular localizations [12, 13]. The *PIN* gene family has eight members in *Arabidopsis* and every member seems responsible for different functions in auxin efflux [14, 15], and the tomato *PIN* gene family expanded to ten members (*SlPIN1*-*SlPIN10*) [16, 17]. Arabidopsis has 29 ATP Binding Cassette subfamily B (ABCB) members, and several of the 21 full-length ABCBs have been shown to transport auxin: AtABCB1 and AtABCB19 [18–21], AtABCB4 [18, 22], AtABCB21 [23], AtABCB6 and AtABCB20 [24]. Tomato also has 29 *ABCBs* with six members grouping with the Arabidopsis auxin tranporter gene family [25]. In Arabidopsis, *AUX1* belongs to a small multigene family comprised of four highly conserved genes (i.e., *AUX1* and *LIKE AUX1* [*LAX*] genes *LAX1*, *LAX2*, and *LAX3*) [26–29], while the tomato *AUX1/LAX* gene family is slightly expanded and contains five members (*SlLAX1*-*SlLAX5*) [17]. These auxin carriers and transporters provide robust functional redundancy and increase auxin flow capacity when needed [30].

While auxin has long been known to regulate AR formation and is routinely used to stimulate root formation in cuttings, interactions with other hormones and overall hormone homeostasis have been shown to be important in lateral root development [31–33]. However, some details of AR induction and development are still outstanding. AR development may vary widely among species from recapitulating the well-defined mechanisms of lateral root (LR) induction and growth to regulation via ARF6 and ARF8 and jasmonic acid in *A. thaliana* hypocotyls [34–37]. Understanding this process is critical in order to improve the efficiency and cost of mass propagation of horticultural and forestry plants, some of which are recalcitrant to AR formation, including apple, pear, peach, walnut and chestnut [38–41].

Here we examine the mechanism of AR formation in tomato stem cuttings. AR formation was investigated via the analysis of AR primordia numbers and length under different treatments, changes in phytohormone accumulation, and expression analysis of genes encoding auxin transporters. The results presented here show that auxin positively regulates AR formation at the cellular level.

## Results

### Anatomical observation of AR formation in tomato cuttings

**Fig. 1:**
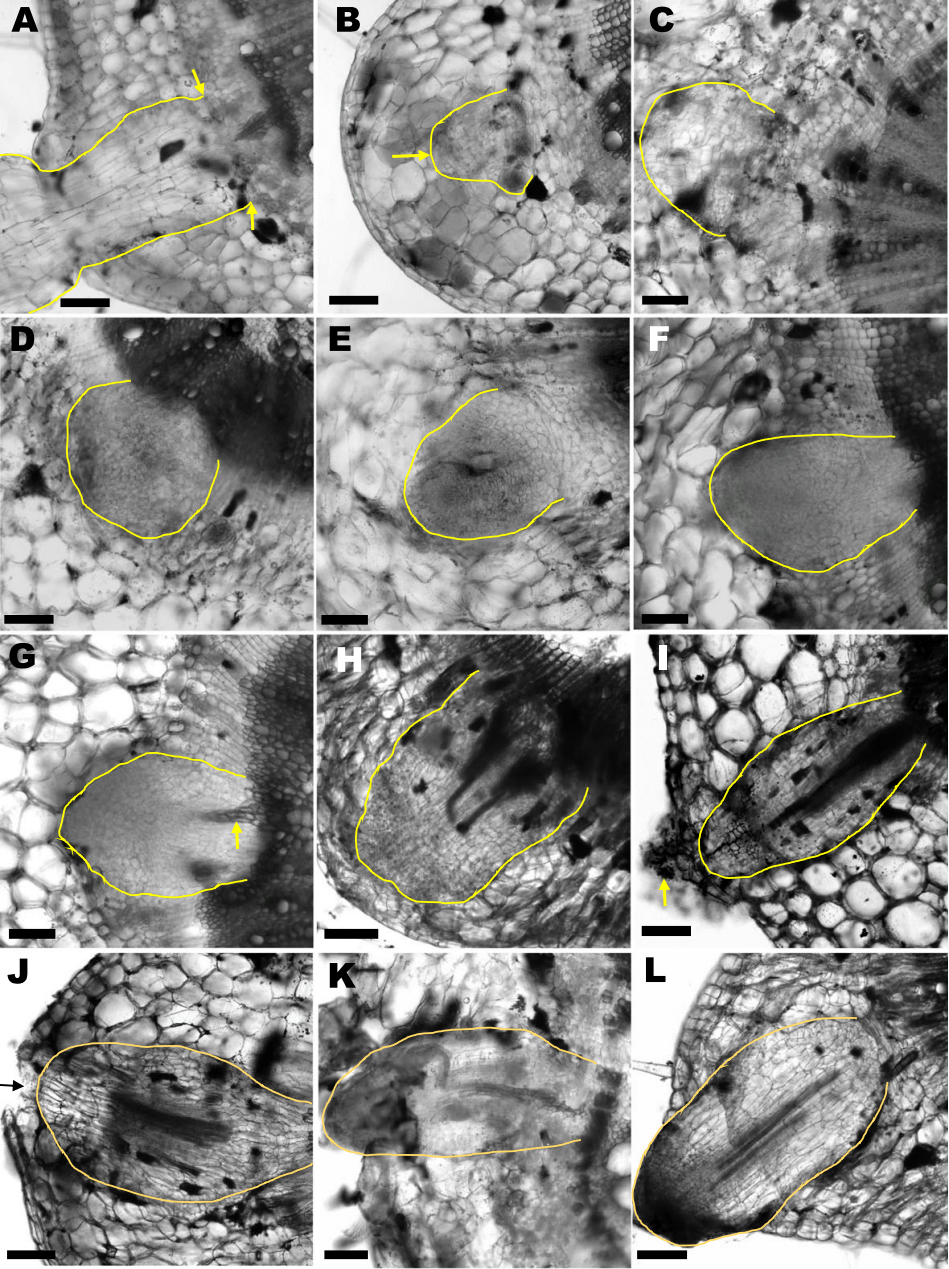
Developmental phases of adventitious root formation in 4-week-old tomato cuttings. Transverse tomato stem cuttings were sectioned to visualize the phases of AR formation. **a** AR formation originated from the stem pericycle cells (arrow) adjacent the endodermis and the vasculature. **b**-**l** Different morphological development phases during tomato AR formation showing the origin of AR. **b** A few cells organized into a cluster. Arrow points to a disordered cell cluster. **c** Expanded cell cluster. **d**-**g** Different AR development phases showing the AR initiation process through the beginning of new vasculature formation. Arrow in (**g**) points to AR vascular tissue formation. **h** Newly formed AR vasculature connecting to the stem vasculature.. (I-J) AR extension to emergence. Arrow points to an extending AR primordium. **k**, **l** Mature AR emerges from the stem. AR are outlined in yellow all images Bars=100 μm

Anatomical changes that occurred during AR formation in transverse sections of tomato cuttings were visualized using differential interference contrast (DIC) microscopy. In tomato stem cuttings, AR originated from pericycle cell layer (PCL) founder cells (Fig. 1a). The founder cells initially organized into a small disordered cell-cluster (Fig. 1b), and then divided and expanded into a larger, but still disordered, cell cluster (Fig. 1c). This cell cluster eventually developed into a dome-shaped AR primordium (Fig. 1d-f). The inward facing cells of the AR primordium were observed to differentiate into vascular tissue (Fig. 1g), which eventually connected to the vasculature of the stem (Fig. 1h) to form a continuous and functional vascular system, presumably via canalization [42, 43]. The outward facing cells of the developing AR continued to divide and elongate, extending through the stem cell layers (Fig. 1i), until the AR emerged through the stem epidermis (Fig. 1j). The AR emergence process damaged the epidermal cells of the stem, which caused the outer stem epidermal layers to slough off. Finally, the mature AR continued to elongate after it emerged from the stem (Fig. 1k, l).

### Auxin accumulates above the tomato stem excision site

**Fig. 2:**
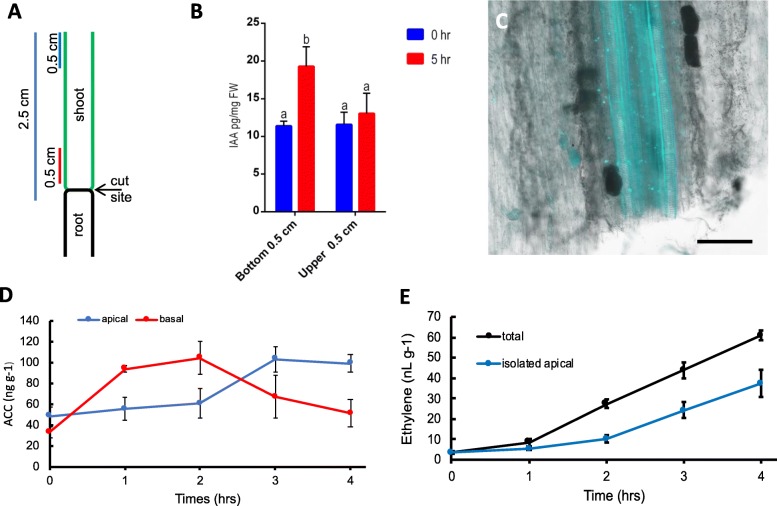
Auxin quantification in hypocotyl sections from 19-day old tomatoes. **a** Cartoon indicating how tissue was collected for auxin determinations. **b** Tomato hypocotyls were excised at the root-shoot transition zone and transferred onto an agar block before being placed into an enclosed vertical mesh transfer box for 5 h (T5). Control samples were not transferred onto agar blocks, but were instead immediately collected and frozen in liquid nitrogen. Auxin levels in the bottom 0–0.5 cm and upper 2–2.5 cm at the base of the excision were quantified via LC-MS/MS. Data are means ± standard deviation, *n* = 3. **c** Confocal laser spectral scanning microscope observation of a tomato cutting 5 h post-excision. Size bar, 2 mm. **d** ACC quantifications were as for auxin quantitations. Data are means ± standard deviation, n = 3. **e** Ethylene quantifications were as for auxin quantitations except the headspace was collect and measured by GC. Data are means ± standard deviation, *n* = 3

Previous studies have shown that local auxin maxima promote AR formation [44]. This led to the hypothesis that auxin pools in the basal stem prior to AR formation [45–47]. Previous studies in petunia [48] and pea [49] also showed that auxin levels increased and peaked in cut stems post-excision and subsequently decreased. To test this hypothesis in tomato, IAA levels were quantified at the base of 19-day old tomato stem cuttings. Tomato plants were excised at the root-shoot transition zone to produce the stem cutting. Half-centimeter sections were collected from the base of the explant at 0-, 1- and 5-h post-excision (hpe) (Fig. 2a). Auxin levels were quantified via liquid chromatography with tandem mass spectrometry (LC-MS/MS). The data showed that more IAA accumulated in the bottom 0–0.5 cm of excised stems than the upper 2–2.5 cm basal stem at 5 h (Fig. 2b; *P* < 0.05). In contrast, there were no differences in IAA levels between bottom 0–0.5 cm and upper 2–2.5 cm samples from control plants (Fig. 2b). These results suggest that once a tomato stem has been excised, auxin pooled approximately 0.5 cm above the cut site over 5 h. The removal of the sink root tissue resulted in the deposition of callose at the basal side of vascular tissues directly above the cut site, which is clearly shown by aniline blue staining (Fig. 2c).

Since auxin and ethylene interactions were shown to positively regulate AR in *Arabidopsis* [50], the ethylene precursor aminocyclopropane-1-carboxylic acid (ACC) and ethylene were also measured in the basal and apical stem cutting. ACC accumulation increased in the basal stem from 1 hpe, peaked at 2 hpe and then declined, while in the apical stem ACC increased and peaked at 3 hpe (Fig. 2d). Ethylene levels paralleled ACC levels during the first 3 h and continued to increase over time (Fig. 2e).

### Auxin accumulation patterns during AR formation

**Fig. 3:**
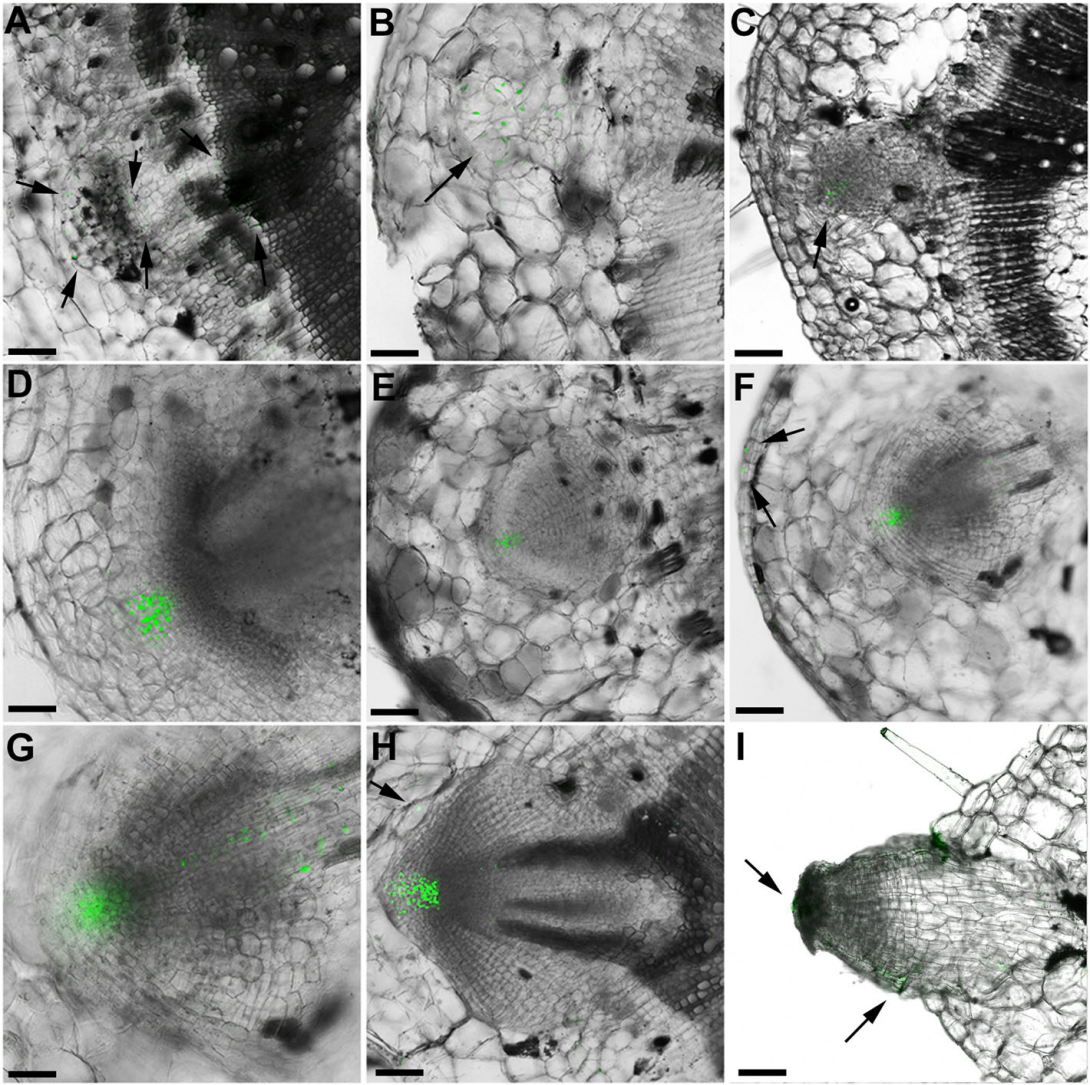
Auxin accumulation patterns during AR formation in tomato plants. Confocal spectral laser scanning microscopy was used to image DR5pro:YFP (green) fluorescence localization during AR development in tomato stem cuttings. **a** Founder cells which arose from pericycle cells. Arrows point to cells with YFP signals. **b** Expanded AR founder cell cluster. Arrow points to cells with YFP signals. **c** Rudimentary AR primordium. Arrow points to cells with YFP signals. **d**, **e** Developing AR primordium. **f** Developing AR primordium vasculature. Arrows point to epidermal cells with YFP signals. **g** Two-fold magnification of (**f**). **h** Mature AR primordium emerging from stem. Arrows point to YFP signals in cell adjacent to AR primordium. **i** Emerged AR. Arrow points to cells with YFP signals. Bars=100 μm

To further investigate the role of auxin during AR formation, transgenic tomato plants expressing the YFP gene under the control of the auxin responsive DR5 synthetic promoter [51, 52] were visualized over a time course of AR development. Figure 3 highlights the developmental phases when and where auxin accumulation was observed using confocal laser scanning microscopy. YFP signals were observed during founder cell initiation in the pericycle cells (Fig. 3a). As the founder cells divided, the YFP signals also expanded throughout the disordered cell cluster (Fig. 3b). In the rudimentary AR primordium, YFP signals were mainly observed in the developing root tip, suggesting that a high auxin concentration is required at this developmental stage (Fig. 3c, Additional file 5: Figure S1A, B). During early stages of AR primordium growth and development, YFP was mainly localized in the AR apical meristem (Fig. 3d, e), and then expanded to the developing vasculature and epidermal cells closest to the AR meristem (Fig. 3f, g). YFP signals were also observed in apical cells of the mature AR primordium (Fig. 3h). Finally, AR vascular tissue developed and then connected to the main stem vasculature tissue to form a continuous vasculature (Fig. 3h). At this time, strong YFP signals were observed in the apex of AR primordium and adjacent cells, suggesting that the newly developed AR represents a novel sink for auxin transport from the main stem. Finally, after AR emergence, YFP signals were observed in the apical meristem and in the presumptive AR elongation zone (Fig. 3i, Additional file 5: Figure S1C).

### Auxin distribution in developing LR and AR

**Fig. 4:**
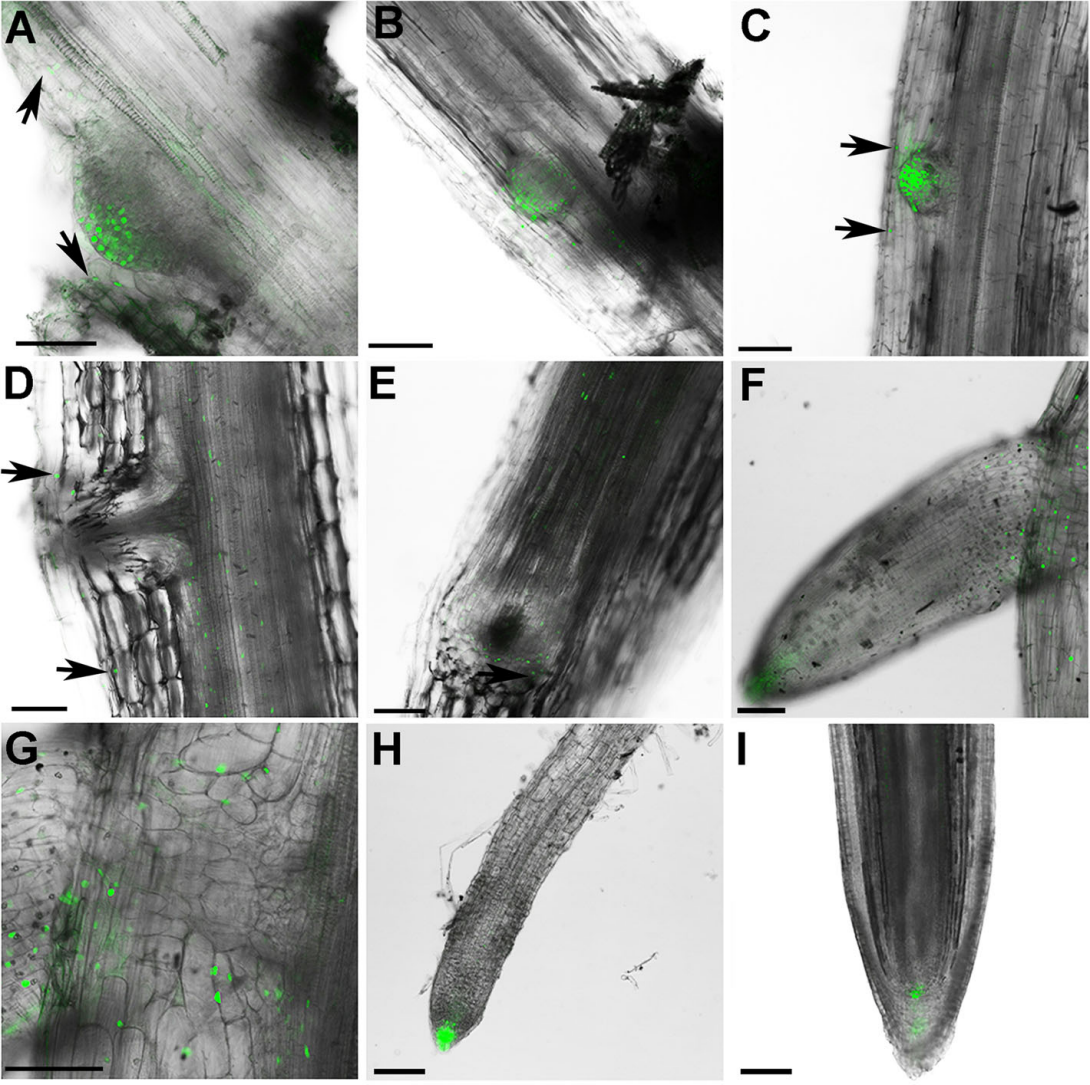
Auxin accumulation patterns during LR formation in transgenic tomato plants. Confocal spectral laser scanning microscopy was used to image DR5pro:YFP (green) fluorescence localization during LR development in tomato roots. **a** LR initiation, during which several cells from pericycle layers have differentiated into a small dome shape. **b**, **c** Developing LR primordium. **d** LR primordium vasculature formation. **e** Transverse-section of (**d**) which shows the connection between a developing LR and the primary root. **f** LR emergence from primary root epidermis. **g** Two-fold magnification of (**f**) at the point of emergence from the primary root. **h** Mature LR. **i** Primary root. YFP fluorescence signal is shown in green. **a**, **c**, **d** Arrows point to YFP signals in the primary root adjacent to LR primordium. Bars = 100 μm

Since both AR and LR originate from pericycle cells, auxin distribution during LR formation in tomato cuttings was also examined. In contrast to AR, DR5pro:YFP signals were clearly observed in every cell of the dome-shaped cluster of LR founder cells, in pericycle cells adjacent to the LR initiation site (Fig. 4a, b) and in cells adjacent to the LR primordium (Fig. 4b, c). At maturity, the LR primordium vascular tissue was connected to the vasculature of the main root (Fig. 4d). Interestingly, YFP signals in mature LR were still observed in cells adjacent to the LR (Fig. 4e). In newly emerged LR, YFP signals were observed in the root cap, root stem cell niche, adjacent primary root cells and vasculature (Fig. 4f, g), while the signal was concentrated in the emerged AR root tip (Fig. 3i, Additional file 5: Figure S1C). The auxin distribution gradient in mature LR recapitulated that in the mature primary root, in which a high auxin gradient was localized in the root cap and stem cell niche (Fig. 4h, i).

### Exogenous auxin treatment promotes AR formation in tomato cuttings

**Fig. 5:**
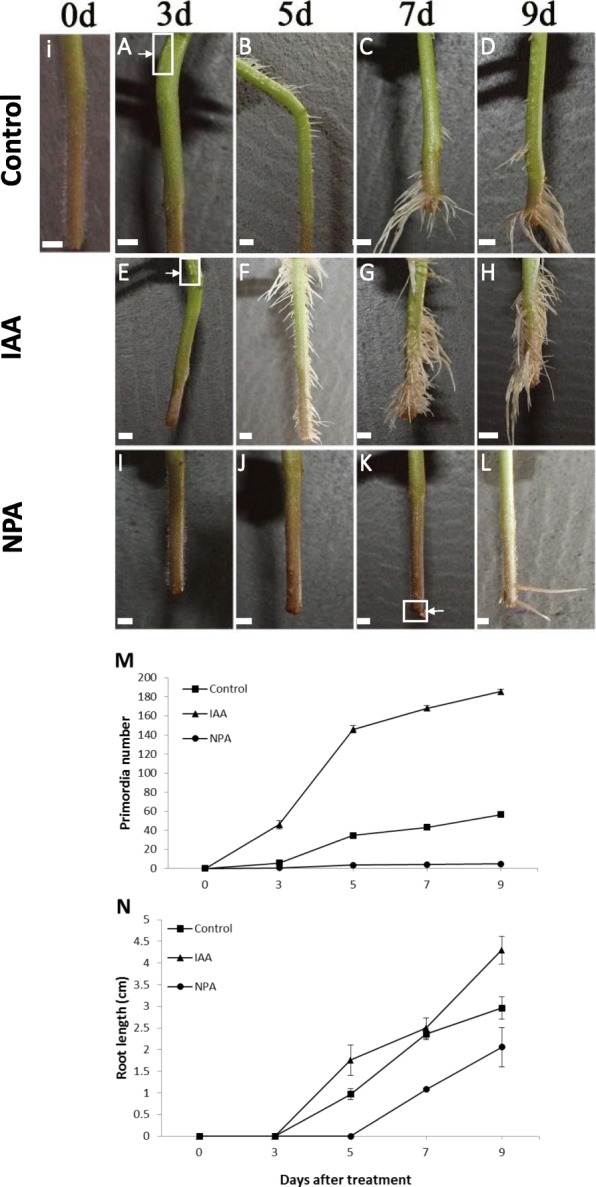
Effects of exogenous IAA and NPA on AR formation in tomato cuttings. Tomato stem cuttings were grown in hydroponic solution to which either 10 μM IAA or 10 μM NPA was added, and AR primordia and roots were observed over a 9d time course. (i) Stem cutting at time 0 (0d). **A**-**D** AR formation in control stems. **E**-**H** AR formation in IAA-treated cuttings. Box and arrowhead in (**A**) and (**E**) show AR primordia. **I**-**L** AR formation in NPA-treated cuttings. Bow and arrowhead in (**K**) show curling root. Bars = 0.5 cm. Primordia number (**M**) and root length (**N**) at different time points of control, IAA- and NPA-treated tomato cuttings. Data are means and standard errors of five plants. The experiment was repeated twice. Data were collected 3, 5, 7 and 9 dpe

Since auxin has been shown to be involved in AR formation, the effects of exogenous auxin treatment on this process were investigated. At the time of stem cutting (0d), no AR primordia were observed (Fig. 5i). Under control conditions, AR primordia could be observed in tomato cuttings 3 days post-excision (dpe) (Fig. 5A). AR maturation was gradual (Fig. 5B), and AR matured into a functional root-system between 7 and 9 dpe (Fig. 5C, D). When IAA was included in the media, the number of AR primordia in 3 dpe cuttings increased to nearly 8-fold that of the control (Fig. 5E, M). In 5 dpe cuttings, AR were visible in control and IAA treatments, and the number and length of AR in IAA-treated cuttings were 4- and 2-fold higher than control, (Fig. 5B, F, M, N). From 7 to 9 dpe, AR in both control and IAA treatments were numerous and elongated sufficiently to form a new root system (Fig. 5C, D, G, H, M, N). In contrast, there were 7 times less AR primordia in cuttings treated with the pre-emergent herbicide and auxin transport inhibitor NPA in comparison to the control cuttings at 3 dpe (Fig. 5A, I, M), and the cuttings remained in an early developmental phase at 5 dpe and occasionally displayed a root curling phenotype (Fig. 5K), which has been described in other species treated with NPA, such as Arabidopsis and maize [53–55]. From 7 to 9 dpe, NPA-treated AR were few, underdeveloped, thick, and agravitropic (Fig. 5K, L).

### Cytokinin, abscisic acid and salicylic acid accumulation during AR formation

In tissue culture, cytokinin and auxin promoted different developmental patterns: higher auxin concentrations induced root formation while higher cytokinin levels induced shoot formation [56]. Furthermore, previous studies revealed crosstalk between abscisic acid (ABA) and IAA in regulating lateral root growth [57, 58]. In addition, a link between salicylic acid (SA) levels and the number of lateral roots has been reported [11]. Therefore, the effects of zeatin (a cytokinin, CK), abscisic acid (ABA) and salicylic acid (SA) accumulations were analyzed in submerged and unsubmerged stems and leaves from tomato cuttings over the time course of AR formation: induction (0 to 72 hpe), initiation (72 to 120 hpe) and extension (120 hpe).

**Fig. 6:**
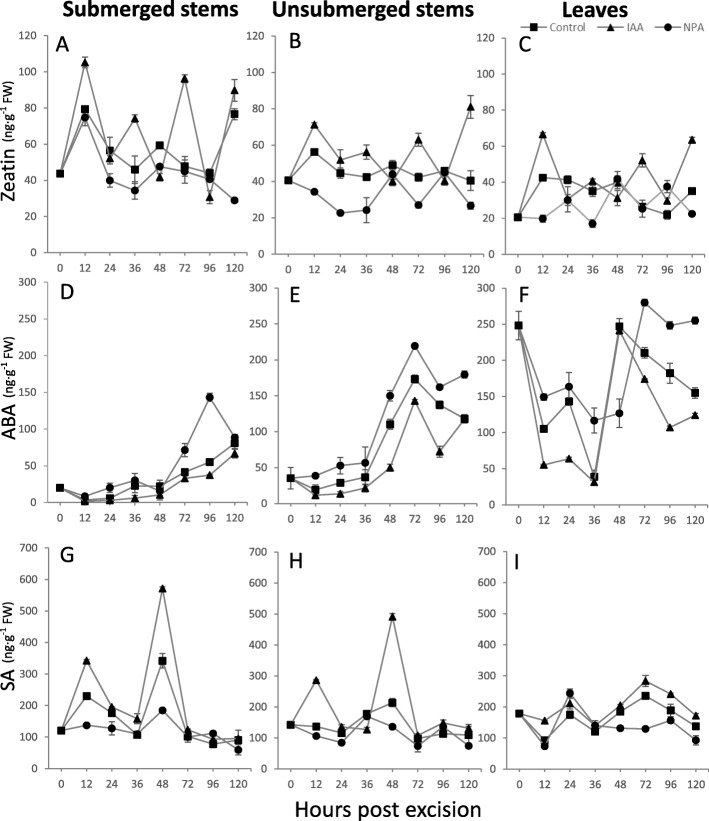
Phytohormone levels observed in tomato cuttings during AR formation. Zeatin (**a**-**c**), abscisic acid (ABA) (**d**, **e**) and salicylic acid (SA) (**g**-**i**) levels were quantified in tomato shoots during AR formation 0–120 h post excision via LC-MS, under the following treatments: control, 10 μM IAA or 10 μM NPA. Hormone levels were determined in shoots: submerged and unsubmerged stems, and leaves for each treatment. Data are means and standard errors, of 5 shoots, and the experiment was repeated 2 times. Different letters in the same index means the significant difference among samples at each time point under the control, NPA as well as IAA, separately (*P*< 0.05)

Zeatin levels were highest in the submerged portion of stems followed by the unsubmerged portion of stems and then leaves under control conditions over the time course analyzed (Fig. 6a-c). Submerged stems showed highest zeatin levels at 12 hpe, with a second peak at 120 hpe, corresponding to the AR induction and extension phases, respectively. When the submerged stems were treated with IAA, the pattern of zeatin accumulation was similar to controls, but more zeatin was measured in all tissues and additional peaks were observed at 36 and 72 hpe, corresponding to the AR initiation phase. Initial zeatin levels in the NPA-treated submerged stems did not differ from the control or IAA-treated stems until 24 hpe through the end of the experiment, and the zeatin levels were reduced in the NPA-treated cuttings. The zeatin levels in control and IAA-treated unsubmerged stems and leaves followed the general pattern of submerged stems, but with less overall zeatin levels (Fig. 6b, c). Zeatin levels in NPA-treated unsubmerged stems and leaves were less than controls from 12 hpe through the end of the time course. These results suggest that auxin-cytokinin interactions rather than absolute levels of these hormones regulate AR development.

ABA accumulation in tomato cuttings was the inverse of zeatin levels. Leaves accumulated the highest amount of ABA, followed by unsubmerged stems and then submerged stems (Fig. 6d-f). Initial ABA levels were low in unsubmerged and submerged stems (0 through 36 hpe). At 48 hpe, ABA levels increased in unsubmerged and submerged stems, but did not approach the high ABA levels of leaves. When unsubmerged and submerged stems were treated with IAA, ABA levels followed the same trend as observed in the respective control. NPA treatment increased ABA levels in unsubmerged from 72 to 96 hpe and submerged stems from 12 to 120 hpe compared to the respective control and IAA treatments. The ABA levels in leaves were high at the time of cutting, and decreased at 12 hpe where it plateaued and then decreased at 36 hpe. ABA levels increased in IAA- and NPA-treated leaves at 48 hpe followed by a decline in levels, and in controls at 72 hpe, where ABA levels remained steady through the end of the experiment. It appears that the ABA accumulation maximum occurs in tomato leaves under normal growth conditions, and can be attenuated by IAA and NPA, especially in the early phase of AR formation.

SA levels were highest in submerged and was unsubmerged stems and lowest in leaves (Fig. 6g-i). SA levels in submerged stems showed peaks at 12 and 48 hpe in control and IAA treatment, and SA levels were low in NPA-treated stems (Fig. 6g). SA levels in IAA-treated unsubmerged stems showed peaks at 12 and 48 hpe (Fig. 6h). SA levels in leaves were low, but showed small peaks aat 24 and 72 hpe in control and IAA-treated leaves (Fig. 6i). The peak level of SA at 12 hpe and 48 hpe in submerged stems correlates with AR primordia initiation. Together, these data suggest that hormone homeostasis is important during all AR stages.

### Expression of auxin transporters

**Fig. 7:**
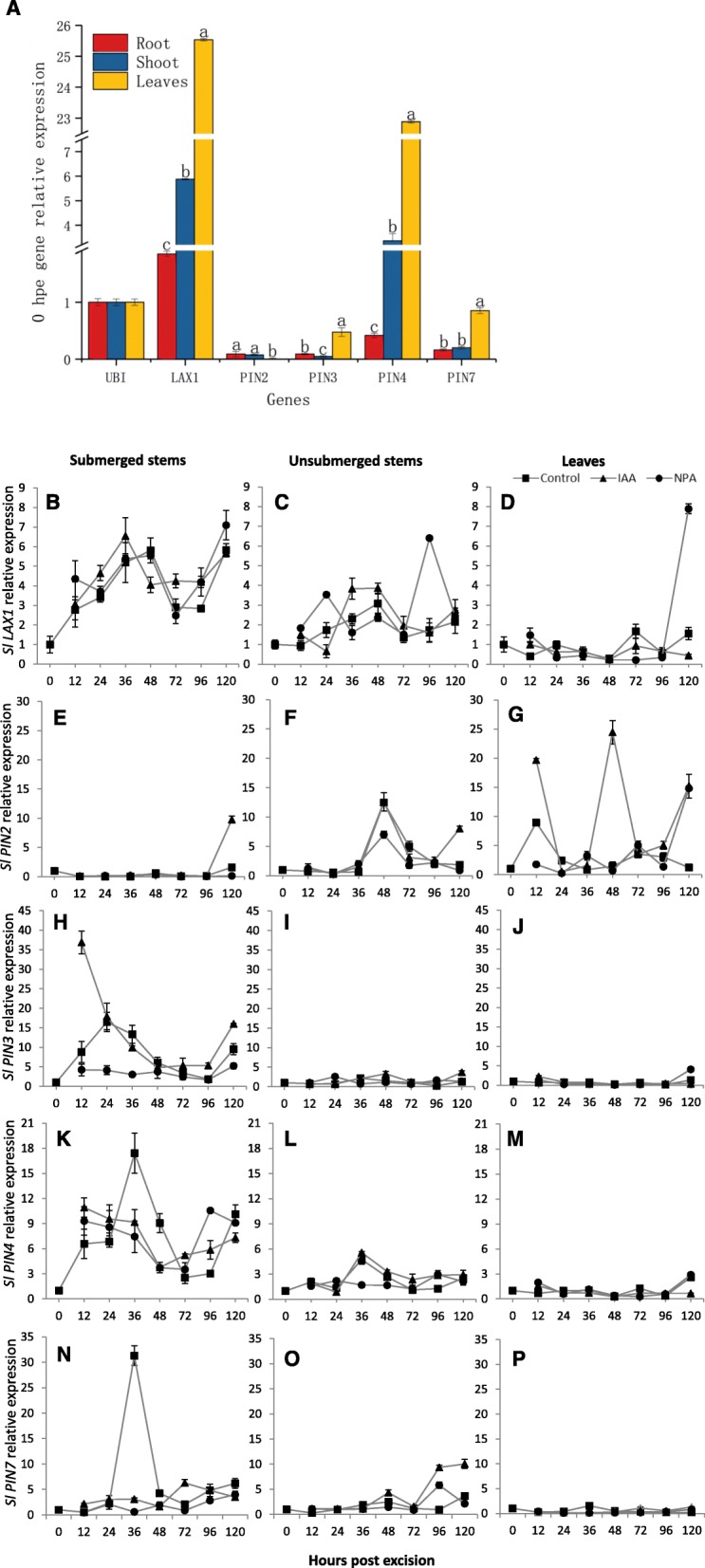
Expression of genes encoding auxin symporters and carriers during AR formation. **a** Quantitative real-time PCR (qRT-PCR) gene expression profiles of tomato plasma membrane auxin transporter genes in tomato. The relative expression of each gene (arbitrary units) corresponds to gene expression normalized to *SlUBI3* expression. Roots, stems and leaves were collected from whole plants for analyses. **b**-**p** qRT-PCR gene expression profiles of auxin symporters and carriers during AR development in tomato shoot cuttings: submerged and unsubmerged stems, and leaves for each treatment. Relative expression was measured via qRT-PCR in leaves, and 5-mm segments, which were cut from the submerged and unsubmerged stems, respectively. The relative expression of each gene (arbitrary units) corresponds to gene expression normalized to *SlUBI3* expression, and 0 hpe was set to 1. Bars represent the standard deviation (*n*=3)

Since auxin treatments had the greatest effect on AR formation (Fig. 5), it was hypothesized that auxin transport to areas of AR induction, initiation and emergence was crucial. To test this hypothesis, expression of genes encoding auxin symporters and carriers was examined in tomato plants (Fig. 7a) and shoots (Fig. 7b-m) via quantitative real-time PCR.

The tomato AUX/LAX homologue, *SlLAX1*, was strongly expressed in roots, stems and leaves with the highest expression level detected in leaves (Fig. 7a). *SlLAX1* expression in submerged stems showed peaks at 36 and 120 hpe in control, and IAA and NPA treatments (Fig. 7b), corresponding to AR induction and extension phases. In unsubmerged stems, the peak expression was at 48 hpe in controls, 36–48 hpe in the IAA treatment and 24 and 96 hpe in the NPA treatment (Fig. 7c). *SlLAX1* expression was low in leaves in the control and treatments, although there was a peak at 120 hpe in NPA-treated leaves (Fig. 7d). These patterns suggest that there are discrete increases in *SlLAX1* expression at each of the three stages of AR development in stems.

Four *PIN* family members were examined and showed differential expression patterns. *SlPIN2* was expressed at low levels in roots, stems and leaves (Fig. 7a), while *SlPIN3* expression was low in roots and stems and significantly greater in leaves (Fig. 7a). *SlPIN4* was highly expressed in leaves, followed by stems with low expression in roots. *SlPIN7* expression pattern was similar to *SlPIN3* (Fig. 7a).

*SlPIN2* was expressed at low levels in submerged stems, with a 2-fold increase at 120 hpe, which was nearly 9-fold upon IAA treatment compared to 0 hpe (Fig. 7e). Unsubmerged stems showed a 10-fold increase in *SlPIN2* expression at 48 hpe in control and the treatments compared to 0 hpe (Fig. 7f). In leaves, *SlPIN2* expression showed a peak at 12 hpe and increased significantly at 12, 48 and 120 hpe in the IAA treatment and 120 hpe in NPA treatment (Fig. 7g). Therefore, the expression of *SlPIN2* expression increased during AR induction and extension phases in shoots.

*SlPIN3* expression increased from 12 hpe through 36 hpe in submerged stems, and then again at 120 hpe (Fig. 7h). *SlPIN3* expression also increased at these time points in IAA-treated stems, while stems treated with NPA showed decreased expression at 24 and 36 hpe (Fig. 7h). In unsubmerged stems and leaves, *SlPIN3* expression was low in control and the treatments (Fig. 7i, j). *SlPIN3* was most highly expressed during the induction phase in unsubmerged stems.

*SlPIN4* expression increased 10-fold at 36 hpe in control submerged stems, and expression in IAA and NPA treatments were largely similar to each other and to control (Fig. 7k). In unsubmerged stems, *SlPIN4* also showed a slight increase at 36 hpe control and IAA treatment (Fig. 7l). In leaves *SlPIN4* expression was low in controls and treatments (Fig. 7m). This suggests that *SlPIN4* may have a role during AR induction in submerged stems.

In submerged stems *SlPIN7* expression increased nearly 20-fold at 36 hpe and then declined (Fig. 7n). IAA or NPA treatment on *SlPIN7* expression had little effect (Fig. 7n) as on *SlPIN4*. In unsubmerged stems, *SlPIN7* expression was low in controls and treatment, and expression increased slightly at 96 and 120 hpe in IAA-treated stems, and NPA-treated stems at 96 hpe (Fig. 7o). In leaves, *SlPIN7* expression was low in the control and treatments (Fig. 7p). This suggests that *SlPIN7* may have a role during AR induction in submerged stems.

## Discussion

### Adventitious and lateral root formation are distinct processes

The mechanism and regulation of AR formation have not been fully characterized, especially in comparison to the extensive knowledge of LR development [59, 60]. Both AR and LR develop from pericycle cells, but the mechanism regarding how one pericyclic cell begins to form AR or LR and another does not, is still unknown. One hypothesis is that the pericycle is “primed” for formation of LR [61, 62] or AR from hypocotyls [63]. However, LR also emerge from root bend regions [64], suggesting that there is more than one mechanism. Adventitious root development appears to follow a developmental program after receiving a stimulus: founder cells organize into a disordered cluster, then gradually form a dome shape that differentiates into an AR primordium. Then cells within the primordium differentiate into vasculature tissue that eventually connects to the stem vasculature (Fig.1a-h), thus allowing AR to become functional roots. In the final step, the primordium emerges from stem epidermis resulting in a mature AR (Fig. 1k, l). The stem epidermal cells undergo programmed cell death (PCD) which allows the AR to emerge [9, 65]. In general, a timeline of AR formation can be mapped based on these observations: AR induction occurred between 0 to 3 dpe, AR initiation between 3 to 5 dpe, and AR extension and emergence from the stem at 5 dpe.

In contrast to the PCD observed during AR development, auxin induces expression of cell wall remodeling enzymes which results in cortical and epidermal separation to allow the elongating LR to emerge without causing cell death [29, 66, 67]. One hypothesis for the observed differences between AR and LR emergence is that the cell walls of root epidermal cells can be remodeled to allow LR emergence, whereas stem epidermal cells are not easily remodeled, therefore PCD is required for AR emergence. While PCD of the epidermal cells occurs during AR emergence, cell wall remodeling during AR development and elongation is also likely.

Previous reports also suggested that PCD is triggered by the interaction between ethylene and auxin at the base of the plant [9, 65]. This is consistent with the increased ACC (ethylene precursor), ethylene and auxin accumulation observed at the base of cut stems (Fig. 2) and suggesting potential crosstalk between the two hormone signaling pathways. This is consistent with previous data showing that wounding induced a local increase in ethylene levels, which in turn promoted AR emergence [68]. In Arabidopsis, addition of ACC to auxin treatments enhanced AR, while ACC alone did not [50]. Overall, ethylene appears to be a negative regulator of lateral root formation [69], while it is a positive regulator of tomato AR development via initiation of AR and promotion of AR emergence.

### Auxin responses are similar during AR and LR formation

The DR5 reporter has been used to examine auxin gradients during plant development [70, 71] and it is a useful tool to examine AR formation. DR5pro:YFP signals were detected in almost every cell in the earliest developmental phase of the AR cell cluster, and signals were subsequently confined to the apical region. During maturation, YFP was restricted in three areas of the root stem cell niche: root cap and adjacent cells, developing vasculature (Fig. 3) and the stem cell niche (Additional file 5: Figure S1). While there are some differences in YFP expression domains during AR development compared to LR development, overall auxin gradients in AR development were similar to those observed in the primary and lateral roots (Figs. 3 and 4, Additional file 5: Figure S1).

### Roles of IAA, zeatin, ABA and SA in tomato AR development

Previous studies showed that auxin and polar auxin transport are crucial for AR formation [46, 72, 73], and this study examined auxin levels and the expression of genes encoding auxin transporters in AR formation. IAA treatment stimulated AR primordia formation and elongation (Fig. 5E-H, M, N). Moreover, the rate of primordia initiation and elongation was enhanced by IAA treatment. These data suggest that IAA stimulates founder cells for AR primordium initiation. The effects of auxin on AR elongation appear to be secondary compared to primordium initiation because at 168 hpe the length of AR were the same in the control and auxin-treated cuttings (Fig. 5N). It is also possible that 168 hpe, stems no longer respond to IAA to induce primordium initiation.

Not surprisingly, treatment with the pre-emergent herbicide and PAT inhibitor NPA blocked AR formation and elongation at all developmental stages (Fig. 5I-L, M, N), consistent with previous studies [48, 74]. PAT inhibition has also been shown to cause re-localization of the auxin maxima, resulting in associated changes in patterning and polarity [70, 75]. When PAT was blocked by NPA, not only was AR formation delayed, but additional abnormal developmental defects, including thick AR and loss of gravitropism, were observed (Fig. 5E-H), similarly to what was observed in maize tillers [55] or Arabidopsis roots [53, 54].

Cytokinins positively regulate cell division and root length elongation [76, 77], as well as post-embryonic root development. Studies have shown that cytokinin inhibited LR initiation and stimulated LR elongation [78, 79]. Here, zeatin concentrations peaked early (12 hpe) in tomato cuttings (Fig. 6), perhaps due to zeatin induction by the initial wounding. After 12 hpe, zeatin levels decreased until after AR primordium initiation. Subsequently, the zeatin levels were still 2-fold higher than the baseline (0 hpe). These results suggest that cytokinin may positively promote AR extension, and might negatively regulate AR primordium initiation, similar to the function of cytokinin during LR development [74, 80], and consistent with the different functions of auxin and cytokinin observed in tissue culture studies [56, 81].

ABA is typically induced during environmental stress as part of an adaptation mechanism [82, 83]. AR formation is usually induced under stress conditions such as natural flooding or during horticultural/ornamental asexual propagation via cutting and rooting [8]. In all treatments, ABA levels were highest in leaves, perhaps due to drought stress induced by cutting off the root. Therefore, ABA levels were highest in non-rooting tissues. IAA-treated stems had the lowest ABA and greatest number of AR, while NPA-treated stems contained the highest ABA levels and lowest number of AR (Fig. 5). Previous studies in rice showed that ABA indirectly negatively regulated AR formation via inhibition of ethylene-induced PCD and gibberellic acid-promoted PCD [84, 85]. These results suggest that IAA may attenuate ABA levels, so that PCD required for AR emergence can proceed. In contrast, NPA treatment increased ABA where almost no AR emergence and therefore almost no PCD was observed.

Salicylic acid (SA) was shown to positively regulate AR initiation as well as auxin-responsive gene expression and mitotic processes in tomato [8, 86]. Studies in mung bean seedlings showed that SA promoted AR formation via reactive oxygen species [hydrogen peroxide (H_2_O_2_)] accumulation in a dose- and time-dependent manner [87], and more reaction oxygen species are observed in regions of auxin accumulation [88]. SA levels increased 12–48 hpe in all tissues tested, and IAA-treatment enhanced the increase in stems (Fig. 6). SA levels returned to baseline between 72 and 120 hpe in all tissues, when AR primordia are visible (72 hpe), suggesting that SA may promote the AR initiation phase. Since SA is a stress-induced phytohormone [89, 90], excision (wounding) may have induced high SA accumulation in stems in the first 48 h. The sharp decrease in SA levels in stems at 72 hpe further supports that hypothesis.

### Auxin carriers and symporters mainly function during induction and extension phases of AR formation

Auxin transport has been associated with the rooting ability of tomato stem cuttings [8, 91]. Here, the time course gene expression data of submerged stems showed auxin carriers and symporters relative expression generally increased during induction and/ or extension phases (Fig. 7) and suggest that the timing of auxin carrier and symporter expression is critical for AR formation. The results here also show that IAA-treatment increased the expression of auxin carriers and symporters but only when expression increased in controls as well, consistent with auxin treatment increasing AR numbers. A recent study showed that inhibition of basipetal auxin transport by the competitive PAT inhibitor and weak auxin 2,3,5-triiodobenzoic acid (TIBA) reduced the appearance of AR primordia in the tomato mutant *aerial roots* (*aer*), which exhibits profuse and precocious formation of adventitious root primordia along the stem [92]. The effects of the pre-emergent herbicide and PAT inhibitor NPA on gene expression in stems was either similar to controls or IAA treatment, except for *SlPIN3* insubmerged stems when expression was reduced during AR induction (Fig. 7). Previous studies have shown differential NPA sensitivity in *pin3–3* Arabidopsis mutants depending on the process measured [30, 93], pointing to NPA regulation of multiple processes [94]. *SlLAX1* plays a major role in AR development, and is discretely expressed during all three phases of AR. *SlPIN3*, *SlPIN4* and *SlPIN7* appear to be important for AR induction, while *SlPIN2* appears to be important for AR induction and emergence. Therefore, it appears that the two critical stages in AR formation are induction and emergence. Thus, we propose that IAA is channeled via the various auxin carriers and transporters to promote morphogenesis and development of founder cells during AR formation.

## Conclusion

AR formation in tomato stem cuttings is a series of events following perception of a wounding stimulus. Auxin and ACC accumulated above the cut site at the base of the excised tomato stem, and ethylene levels increased in the stem. Similar to LR, AR originated in the pericycle at the base of the cut stem, and DR5pro:YFP signals were detected in almost every cell in the earliest AR developmental phase. Gene expression time course studies suggested that auxin carriers and symporters may play a crucial role in delivering auxin to AR induction and initiation sites. In addition to auxin, this study also showed that ABA, zeatin and SA may play a complementary role in the induction, initiation and emergence of the developing AR. Taken together, these data suggest that upon wounding perception, the tomato shoot stem undergoes a series of time-sensitive biological processes that include changes in gene expression, cellular auxin accumulation, cell division and programmed cell death.

## Methods

### Plant material and growth conditions

Tomato seeds Alisa Craig (AC) and DR5pro:YFP transgenic lines (originally obtained from Thompson and Morgan https://www.thompson-morgan.com/) were surface sterilized for 10 min in 50% (v/v) bleach and then washed twice in double-distilled water. The seeds were then plated on petri dishes containing wet filter paper. The plates were cold treated for 2 days at 4 °C in the dark to synchronize germination. After 4 weeks of growth the shoots were excised and then transplanted into potted soil. The basal 4 cm of cuttings were buried in soil or submerged in hydroponic Hoagland’s solution. The basal portion of stems used in the anatomical observations was continually cultured in pots. The shoots in pots were cultured in the greenhouse with routine management. For the NPA and IAA treatments, tomato shoots were cultured in ventilated hydroponic equipment, which contained Hoagland's nutrient solution at pH 5.8 [95]. The growth conditions of hydroponic equipment were under the photoperiod of light/16 h and dark/8 h, light intensity of 300 μmol m^− 2^ s^− 1^, and temperature of 25 °C, and with 0.5 h ventilation every 2 h. For auxin and 1-aminocyclopropane-1-carboxylic acid (ACC) and ethylene quantitation, tomato seeds from the Alisa Craig (AC) accession were sown on wet soil in pots and incubated in a 4 °C cold-room for 3 days. After the cold treatment, the pots were transferred to a growth room under continuous white-light where the tomato plants grew for nineteen days.

### Chemicals and reagents

IAA and NPA were purchased from Sigma-Aldrich and dissolved in dimethyl sulfoxide. In hydroponic experiments, Hoagland’s media was purchased from Sigma-Aldrich. Modified Hoagland’s media (Sigma - Aldrich, St. Louis MO USA) was used as the hydroponic medium for tomato cuttings. The working concentrations for IAA and NPA treatments were set at 10 μM. Zeatin, ABA and SA and ACC standards for mass spectroscopy were obtained from Sigma-Aldrich.

### Analysis of growth and development

AR length was determined from digital images of each cutting by measuring from root tip to cutting base using ImageJ 1.40 software (http://rsb.info.nih.gov/ij/). The number of AR primordia were counted using magnifying binoculars.

### Callose staining

Callose staining of excised tomato cuttings was performed following the method described by Schenk and Schikora [96]. Before staining, tomato stems were excised at the root-shoot transition zone and kept immersed in double distilled water in a covered petri dish for 5 h. After that, the stems were cut longitudinally using a sharp razor blade. Images were collected using LSM 710 Laser Spectral Scanning Confocal Microscope (Zeiss) using 405 nm excitation, 410–585 nm emission, pinhole set to 600 μm, EC Plan Neofluor 10x/0.30 M27 objective. All images were taken under the same conditions.

### Microscopy

The tomato stems were collected over a time-course of AR formation from 0 h through 120 h. The stems were cut into ~ 100–200 μm transverse sections along the longitudinal axis by hand and then mounted on microscope slides in water. DR5pro:YFP localization was visualized using an LSM 710 Laser Spectral Scanning Confocal Microscope (Zeiss) with EC Plan Neofluor 10x/0.30 M27, Plan Apochromat 20x/0.8 M27 or C-Apochromat 40x/1.20 W Kott M27 water immersion lens, pixel dwell time of 1.58 μs. The master gain was always set to less than 893, with a digital gain of 1.0–1.5. YFP acquisition was in lambda mode, 514 nm (5–10%) excitation and 523 to 573 nm emission were, and the pinhole was set to 36 μm. All of images were taken under the same conditions. All captured images were processed with ZEN Lite 2012 (Zeiss; www.zeiss.com) and Photoshop (Adobe; www.adobe.com).

### Phytohormone assays

To determine zeatin, ABA and SA levels during tomato shoots, 5-mm segments were excised from the base of tomato stems. The segments were placed into ice-cold uptake buffer (1.5% sucrose, 23 mM MES-KOH, pH 5.5,) for 15 min and washed twice in fresh uptake buffer for 15 min. Segments were surface dried on filter paper. Dry segments were weighed. Zeatin, ABA and SA were all extracted from crude tomato shoots and quantified based on a method described by Pan et al. [97] with little modification. For ACC measurements, tomato shoots were grown in a glass jar containing either a gas-permeable or impermeant plastic disk sealed with beeswax and 0.5 cm agar media at the bottom of the jar. ACC was extracted from the apical 1 cm of the stem (with leaves removed) and first 1 cm section (basal) above media. Headspace ethylene was also collected in this same system. Headspace sampling made in split jars, and headspace gas collected by syringe at time points indicated. ACC was AccQ Tag by HPLC (Waters) was measured in SRM/MRM with genuine standards of ACC, MACC, GACC. Ethylene was measured by gas chromatography via HP 6890 gas chromatograph (Agilent) equipped with a Flame from Detector (FID). Ethylene was measures in the total stem and the apical stem.

Hormone levels were quantified from 5 biological replicates using HPLC–ESI–MS/MS Agilent 6460 Triple Quadrupole Dual Mass Spectrometer. Reverse-phase HPLC gradient parameters and selected reaction monitoring conditions for protonated or deprotonated plant hormones ([M + H] + or [M − H] −) are listed in Additional file 1: Table S1 and Additional file 2: Table S2.

For auxin extraction and quantitation in tomato shoots, the method described in Zhang et al. [98], was used. Auxin was extracted from three biological samples (*n* = 3). Each biological replicate was composed of three pooled stem sections. Fisher’s LSD statistical analysis was performed (*p*-value < 0.05).

### Real time PCR

Total RNA was extracted using CTAB reagent and 1 μL was used for cDNA synthesis with the Primer Script TM RT reagent Kit (Taraka Bio, Daliang, China). qRT-PCR was performed using an ABI PRISM 7900HT instrument (Applied Biosystems, http://www.appliedbiosystems.com/) using 5 μL of 10-fold diluted cDNA, 1× SYBR green master mix (Applied Biosystems TM, A25780 CA, USA), and 1 mol/L each of target gene-specific primers (Additional file 3: Table S3, Additional file 4: Table S4) in a final volume of 15 μL. Primer efficiency was tested by standard curve analysis using serial dilutions of a known amount of template and their specificity was confirmed by applicant sequencing. The thermal cycling regime consisted of 2 min at 50 °C, 10 min at 95 °C, followed by 40 cycles of 15 s at 95 °C, 30s at 54 °C, and 30s at 72 °C. Disassociation curves and gel electrophoresis verified amplification of a single product. CT values were calculated using SDS2.1 software (Applied Biosystems) and data was analyzed using the delta delta CT method with *SlUBI3* as a reference gene for normalization [99]. Primers are presented in Additional file 3: Table S3.

## Additional files

Additional file 1: **Table S1.** Reverse-phase high-performance liquid chromatography (HPLC) gradient parameters in mobile phases. (DOCX 13 kb)

Additional file 2: **Table S2.** Conditions used for protonated or deprotonated plant hormones of Zeatin, ABA and SA ([M + H]^+^ or [M − H]^−^). (DOCX 14 kb)

Additional file 3: **Table S3.** Accession numbers of the genes used in this study. (DOCX 13 kb)

Additional file 4: **Table S4.** Primers for qRT-PCR. (DOCX 14 kb)

Additional file 5: **Figure S1.** The detailed elaboration of DR5pro:YFP localization in initiation phase of developing AR. (DOCX 301 kb)

## Data Availability

All data generated or analysed during this study are included in this published article and its supplementary information files.

## Abbreviations

ABA: Abscisic acid
ABC: ATP-binding cassette
AC: Alisa Craig
AR: Adventitious root
AUX/LAX: AUXIN1/LIKE-AUX1
CK: Cytokinin
DIC: Differential interference contrast
DR5: Auxin response element (AuxRE)
IAA: Indole-3-acetic acid
LC-MS-MS: Liquid chromatography with tandem mass spectrometry
LR: Lateral root
MDR: Multidrug resistance
NPA: N-1-naphthylphthalamic acid
PCL: Pericycle cell layer
PGP: Phosphoglycoprotein
SA: Salicylic acid

## References

[1] Birnbaum KD (2016). How many ways are there to make a root?. Curr Opin Plant Biol.

[2] Jr FTD, Hartmann HT. The physiological basis of adventitious root formation. Int Symp Veg Propagation Woody Species. 1988;(227, 227):113–20.

[3] Lucas M, Swarup R, Paponov IA, Swarup K, Casimiro I, Lake D, Peret B, Zappala S, Mairhofer S, Whitworth M (2011). Short-Root regulates primary, lateral, and adventitious root development in Arabidopsis. Plant Physiol.

[4] Zhang Q, Visser EJ, de Kroon H, Huber H (2015). Life cycle stage and water depth affect flooding-induced adventitious root formation in the terrestrial species Solanum dulcamara. Annals Bot.

[5] Li S-W, Xue L, Xu S, Feng H, An L (2009). Mediators, genes and signaling in adventitious rooting. Bot Rev.

[6] Itoh JI, Nonomura KI, Ikeda K, Yamaki S, Inukai Y, Yamagishi H, Kitano H, Nagato Y (2005). Rice plant development: from zygote to spikelet. Plant Cell Physiol.

[7] Jasik J, De Klerk GJ (1997). Anatomical and ultrastructural examination of adventitious root formation in stem slices of apple. Biol Plant.

[8] Guan L, Murphy AS, Peer WA, Gan L, Li Y, Cheng Z-M (2015). Physiological and molecular regulation of adventitious root formation. Crit Rev Plant Sci.

[9] Steffens B, Kovalev A, Gorb SN, Sauter M (2012). Emerging roots alter epidermal cell fate through mechanical and reactive oxygen species signaling. Plant Cell.

[10] Pacurar DI, Perrone I, Bellini C (2014). Auxin is a central player in the hormone cross-talks that control adventitious rooting. Physiol Plant.

[11] Armengot L, Marques-Bueno MM, Jaillais Y (2016). Regulation of polar auxin transport by protein and lipid kinases. J Exp Bot.

[12] Adamowski M, Friml J (2015). PIN-dependent auxin transport: action, regulation, and evolution. Plant Cell.

[13] Bennett T (2015). PIN proteins and the evolution of plant development. Trends Plant Sci.

[14] Paponov IA, Teale WD, Trebar M, Blilou I, Palme K (2005). The PIN auxin efflux facilitators: evolutionary and functional perspectives. Trends Plant Sci.

[15] Wang Y, Chai C, Valliyodan B, Maupin C, Annen B, Nguyen HT (2015). Genome-wide analysis and expression profiling of the PIN auxin transporter gene family in soybean (Glycine max). BMC Genom.

[16] Mounet F, Moing A, Kowalczyk M, Rohrmann J, Petit J, Garcia V, Maucourt M, Yano K, Deborde C, Aoki K (2012). Down-regulation of a single auxin efflux transport protein in tomato induces precocious fruit development. J Exp Bot.

[17] Pattison RJ, Catalá C (2012). Evaluating auxin distribution in tomato (Solanum lycopersicum) through an analysis of the PIN and AUX/LAX gene families. Plant J.

[18] Titapiwatanakun B, Blakeslee JJ, Bandyopadhyay A, Yang H, Mravec J, Sauer M, Cheng Y, Adamec J, Nagashima A, Geisler M (2009). ABCB19/PGP19 stabilises PIN1 in membrane microdomains in Arabidopsis. Plant J.

[19] Noh B, Murphy AS, Spalding EP (2001). Multidrug resistance–like genes of Arabidopsis required for auxin transport and auxin-mediated development. Plant Cell.

[20] Markus G, Kolukisaoglu HÜ, Rodolphe B, Karla B, Joachim B, Beate S, Nathalie F, Zsuzsanna K-K, Csaba K, Robert D (2003). TWISTED DWARF1, a Unique Plasma Membrane-anchored immunophilin-like protein, interacts with Arabidopsis multidrug resistance-like transporters AtPGP1 and AtPGP19. Mol Biol Cell.

[21] Geisler M, Blakeslee JJ, Bouchard R, Lee OR, Vincenzetti V, Bandyopadhyay A, Titapiwatanakun B, Peer WA, Bailly A, Richards EL (2005). Cellular efflux of auxin catalyzed by the Arabidopsis MDR/PGP transporter AtPGP1. Plant J.

[22] Santelia D, Vincenzetti V, Azzarello E, Bovet L, Fukao Y, Düchtig P, Mancuso S, Martinoia E, Geisler M (2005). MDR-like ABC transporter AtPGP4 is involved in auxin-mediated lateral root and root hair development. FEBS letters.

[23] Kamimoto Y, Terasaka K, Hamamoto M, Takanashi K, Fukuda S, Shitan N, Sugiyama A, Suzuki H, Shibata D, Wang B (2012). Arabidopsis ABCB21 is a facultative auxin importer/exporter regulated by cytoplasmic auxin concentration. Plant Cell Physiol.

[24] Zhang Y, Nasser V, Pisanty O, Omary M, Wulff N, Di Donato M, Tal I, Hauser F, Hao P, Roth O (2018). A transportome-scale amiRNA-based screen identifies redundant roles of Arabidopsis ABCB6 and ABCB20 in auxin transport. Nat Commun.

[25] Ofori PA, Mizuno A, Suzuki M, Martinoia E, Reuscher S, Aoki K, Shibata D, Otagaki S, Matsumoto S, Shiratake K (2018). Genome-wide analysis of ATP binding cassette (ABC) transporters in tomato. PloS One.

[26] Péret B, Swarup K, Ferguson A, Seth M, Yang Y, Dhondt S, James N, Casimiro I, Perry P, Syed A (2012). AUX/LAX genes encode a family of auxin influx transporters that perform distinct functions during Arabidopsis development. Plant Cell.

[27] Swarup R, Kargul J, Marchant A, Zadik D, Rahman A, Mills R, Yemm A, May S, Williams L, Millner P (2004). Structure-function analysis of the presumptive Arabidopsis auxin permease AUX1. Plant Cell.

[28] Marchant A (2002). AUX1 promotes lateral root formation by facilitating indole-3-acetic acid distribution between sink and source tissues in the Arabidopsis seedling. Plant Cell Online.

[29] Swarup K, Benková E, Swarup R, Casimiro I, Péret B, Yang Y, Parry G, Nielsen E, De Smet I, Vanneste S (2008). The auxin influx carrier LAX3 promotes lateral root emergence. Nat Cell Biol.

[30] Žádníková P, Petrášek J, Marhavý P, Raz V, Vandenbussche F, Ding Z, Schwarzerová K, Morita MT, Tasaka M, Hejátko J (2010). Role of PIN-mediated auxin efflux in apical hook development of Arabidopsis thaliana. Development.

[31] Ivanchenko MG, Muday GK, Dubrovsky JG (2008). Ethylene–auxin interactions regulate lateral root initiation and emergence in Arabidopsis thaliana. Plant J.

[32] Fukaki H, Tasaka M (2009). Hormone interactions during lateral root formation. Plant Mol Biol.

[33] Stoeckle D, Thellmann M, Vermeer JEM (2018). Breakout—lateral root emergence in Arabidopsis thaliana. Curr Opin Plant Biol.

[34] Gutierrez L, Mongelard G, Floková K, Păcurar DI, Novák O, Staswick P, Kowalczyk M, Păcurar M, Demailly H, Geiss G (2012). Auxin controls Arabidopsis adventitious root initiation by regulating jasmonic acid homeostasis. Plant Cell.

[35] Catherine B, Daniel IP, Irene P (2014). Adventitious Roots and Lateral Roots: Similarities and Differences. AnnuRev Plant Biol.

[36] Verstraeten I, Schotte S, Geelen D (2014). Hypocotyl adventitious root organogenesis differs from lateral root development. Front Plant Sci.

[37] Atkinson JA, Rasmussen A, Traini R, Voß U, Sturrock C, Mooney SJ, Wells DM, Bennett MJ (2014). Branching out in roots: uncovering form, function, and regulation. Plant Physiol.

[38] Velasco R, Zharkikh A, Affourtit J, Dhingra A, Cestaro A, Kalyanaraman A, Fontana P, Bhatnagar SK, Troggio M, Pruss D (2010). The genome of the domesticated apple (Malus× domestica Borkh). Nat Genet.

[39] Arús P, Verde I, Sosinski B, Zhebentyayeva T, Abbott AG (2012). The peach genome. Tree Genet Genomes.

[40] Wu J, Wang Z, Shi Z, Zhang S, Ming R, Zhu S, Khan MA, Tao S, Korban SS, Wang H (2013). The genome of the pear (Pyrus bretschneideri Rehd.). Genome Res.

[41] Nelson CD, Powell WA, Merkle SA, Carlson JE, Hebard FV, Islam-Faridi N, Staton ME, Georgi L: Biotechnology of trees: Chestnut. In: Biology and Biotechnology 656 Pages 2014:1–35.

[42] Bennett T, Hines G, Leyser O (2014). Canalization: what the flux?. Trends Genet.

[43] Mazur E, Benková E, Friml J (2016). Vascular cambium regeneration and vessel formation in wounded inflorescence stems of Arabidopsis. Sci Rep.

[44] Garrido G, Ramón Guerrero J, Angel Cano E, Acosta M, Sánchez-Bravo J (2002). Origin and basipetal transport of the IAA responsible for rooting of carnation cuttings. Physiol Plant.

[45] de Klerk G-J, van der Krieken W, de Jong JC (1999). Review the formation of adventitious roots: new concepts, new possibilities. In Vitro Cell Dev Biol Plant.

[46] Sorin C, Bussell JD, Camus I, Ljung K, Kowalczyk M, Geiss G, McKhann H, Garcion C, Vaucheret H, Sandberg G (2005). Auxin and light control of adventitious rooting in Arabidopsis require ARGONAUTE1. Plant Cell.

[47] Blakesley D. Auxin Metabolism and Adventitious Root Initiation. In: Davis T.D., Haissig B.E. (eds) Biology of Adventitious Root Formation. Springer, Boston: Basic Life Sciences: vol 62, 1994.

[48] Ahkami Amir H., Lischewski Sandra, Haensch Klaus-T., Porfirova Svetlana, Hofmann Joerg, Rolletschek Hardy, Melzer Michael, Franken Philipp, Hause Bettina, Druege Uwe, Hajirezaei Mohammad R. (2008). Molecular physiology of adventitious root formation in Petunia hybrida cuttings: involvement of wound response and primary metabolism. New Phytologist.

[49] Rasmussen A, Hosseini SA, Hajirezaei M-R, Druege U, Geelen D (2014). Adventitious rooting declines with the vegetative to reproductive switch and involves a changed auxin homeostasis. J Exp Bot.

[50] Veloccia A, Fattorini L, Della Rovere F, Sofo A, D’angeli S, Betti C, Falasca G, Altamura MM (2016). Ethylene and auxin interaction in the control of adventitious rooting in Arabidopsis thaliana. J Exp Bot.

[51] Ulmasov Tim, Murfett Jane, Hagen Gretchen, Guilfoyle Tom J. (1997). Aux/IAA Proteins Repress Expression of Reporter Genes Containing Natural and Highly Active Synthetic Auxin Response Elements. The Plant Cell.

[52] Heisler MG, Ohno C, Das P, Sieber P, Reddy GV, Long JA, Meyerowitz EM (2005). Patterns of auxin transport and gene expression during primordium development revealed by live imaging of the Arabidopsis inflorescence meristem. Curr Biol.

[53] Garbers C, DeLong A, Deruere J, Bernasconi P, Söll D (1996). A mutation in protein phosphatase 2A regulatory subunit A affects auxin transport in Arabidopsis. EMBO J.

[54] Zhou H-W, Nussbaumer C, Chao Y, DeLong A (2004). Disparate roles for the regulatory A subunit isoforms in Arabidopsis protein phosphatase 2A. Plant Cell.

[55] McLamore ES, Diggs A, Calvo Marzal P, Shi J, Blakeslee JJ, Peer WA, Murphy AS, Porterfield DM (2010). Non-invasive quantification of endogenous root auxin transport using an integrated flux microsensor technique. Plant J.

[56] Skoog F, Miller C. Chemical regulation of growth and organ formation in plant tissues cultured. In Vitro Symp Soc Exp Biol. 1957. 13486467

[57] Xing L, Zhao Y, Gao J, Xiang C, Zhu J-K (2016). The ABA receptor PYL9 together with PYL8 plays an important role in regulating lateral root growth. Sci Rep.

[58] Seo PJ, Park C-M (2009). Auxin homeostasis during lateral root development under drought condition. Plant Signal Behav.

[59] Jr CJ, Grisafi PL, Fink GR (1995). A pathway for lateral root formation in Arabidopsis thaliana. Genes Dev.

[60] Xuan W, Band LR, Kumpf RP, Van DD, Parizot B, De RG, Opdenacker D, Möller BK, Skorzinski N, Njo MF (2016). Cyclic programmed cell death stimulates hormone signaling and root development in Arabidopsis. Science.

[61] Van Norman JM, Xuan W, Beeckman T, Benfey PN (2013). To branch or not to branch: the role of pre-patterning in lateral root formation. Development.

[62] Xuan W, Audenaert D, Parizot B, Möller BK, Njo MF, De Rybel B, De Rop G, Van Isterdael G, Mähönen AP, Vanneste S (2015). Root cap-derived auxin pre-patterns the longitudinal axis of the Arabidopsis root. Curr Biol.

[63] Sorin C, Negroni L, Balliau T, Corti H, Jacquemot M-P, Davanture M, Sandberg G, Zivy M, Bellini C (2006). Proteomic analysis of different mutant genotypes of Arabidopsis led to the identification of 11 proteins correlating with adventitious root development. Plant Physiol.

[64] Richter GL, Monshausen GB, Krol A, Gilroy S (2009). Mechanical stimuli modulate lateral root organogenesis. Plant Physiol.

[65] Vidoz ML, Loreti E, Mensuali A, Alpi A, Perata P (2010). Hormonal interplay during adventitious root formation in flooded tomato plants. Plant J.

[66] Péret B, Larrieu A, Bennett MJ (2009). Lateral root emergence: a difficult birth. J Exp Bot.

[67] Vilches-Barro A, Maizel A (2015). Talking through walls: mechanisms of lateral root emergence in Arabidopsis thaliana. Curr Opin Plant Biol.

[68] Steffens B, Rasmussen A (2016). The physiology of adventitious roots. Plant Physiol.

[69] Negi S, Sukumar P, Liu X, Cohen JD, Muday GK (2010). Genetic dissection of the role of ethylene in regulating auxin-dependent lateral and adventitious root formation in tomato. Plant J.

[70] Sabatini S, Beis D, Wolkenfelt H, Murfett J, Guilfoyle T, Malamy J, Benfey P, Leyser O, Bechtold N, Weisbeek P (1999). An auxin-dependent distal organizer of pattern and polarity in the Arabidopsis root. Cell.

[71] Benková E, Michniewicz M, Sauer M, Teichmann T, Seifertová D, Jürgens G, Friml J (2003). Local, efflux-dependent auxin gradients as a common module for plant organ formation. Cell.

[72] Ludwig-Müller J, Vertocnik A, Town CD (2005). Analysis of indole-3-butyric acid-induced adventitious root formation on Arabidopsis stem segments. J Exp Bot.

[73] Sukumar P, Maloney GS, Muday GK (2013). Localized induction of the ATP-binding cassette B19 auxin transporter enhances adventitious root formation in Arabidopsis. Plant physiology.

[74] Druege U, Franken P, Hajirezaei MR (2016). Plant hormone homeostasis, signaling, and function during adventitious root formation in cuttings. Front Plant Sci.

[75] Xi W, Gong X, Yang Q, Yu H, Liou YC (2016). Pin1At regulates PIN1 polar localization and root gravitropism. Nat Commun.

[76] Rioukhamlichi C, Huntley R, Jacqmard A, Murray JAH (1999). Cytokinin activation of Arabidopsis cell division through a D-type cyclin. Science.

[77] Werner T, Motyka V, Laucou V, Smets R, Van OH, Schmülling T (2003). Cytokinin-deficient transgenic Arabidopsis plants show multiple developmental alterations indicating opposite functions of cytokinins in the regulation of shoot and root meristem activity. Plant Cell.

[78] Rani DB, Taketa S, Ichii M (2005). Cytokinin inhibits lateral root initiation but stimulates lateral root elongation in rice (Oryza sativa). J Plant Physiol.

[79] Laplaze L, Benkova E, Casimiro I, Maes L, Vanneste S, Swarup R, Weijers D, Calvo V, Parizot B, Herrerarodriguez MB (2007). Cytokinins act directly on lateral root founder cells to inhibit root initiation. Plant Cell.

[80] Zhao Y, Cheng S, Song Y, Huang Y, Zhou S, Liu X, Zhou DX (2015). The Interaction between rice ERF3 and WOX11 promotes crown root development by regulating gene expression involved in cytokinin signaling. Plant Cell.

[81] Hill K, Mathews DE, Kim HJ, Street IH, Wildes SL, Chiang Y-H, Mason MG, Alonso JM, Ecker JR, Kieber JJ (2013). Functional characterization of type-B response regulators in the Arabidopsis cytokinin response. Plant Physiol.

[82] Kumar PP (2013). Regulation of biotic and abiotic stress responses by plant hormones. Plant Cell Rep.

[83] Thalmann MR, Pazmino D, Seung D, Horrer D, Nigro A, Meier T, Kölling K, Pfeifhofer HW, Zeeman SC, Santelia D. Regulation of leaf starch degradation by abscisic acid is important for osmotic stress tolerance in plants. Plant Cell. 2016. 10.1105/tpc.16.00143 PMC5006701 27436713

[84] Sauter M (2005). Epidermal cell death in rice is regulated by ethylene, gibberellin, and abscisic acid. Plant Physiol.

[85] Steffens B, Wang J, Sauter M (2006). Interactions between ethylene, gibberellin and abscisic acid regulate emergence and growth rate of adventitious roots in deepwater rice. Planta.

[86] Raskin I (1992). Salicylate, a new plant hormone. Plant Physiol.

[87] Yang W, Zhu C, Ma X, Li G, Gan L, Ng D, Xia K (2013). Hydrogen peroxide is a second messenger in the salicylic acid-triggered adventitious rooting process in mung bean seedlings. Plos One.

[88] Peer WA, Cheng Y, Murphy AS (2013). Evidence of oxidative attenuation of auxin signalling. J Exp Bot.

[89] Khan MIR, Fatma M, Per TS, Anjum NA, Khan NA (2015). Salicylic acid-induced abiotic stress tolerance and underlying mechanisms in plants. Front Plant Sci.

[90] Cheng F, Lu J, Gao M, Shi K, Kong Q, Huang Y, Bie Z (2016). Redox signaling and CBF-responsive pathway are involved in salicylic acid-improved photosynthesis and growth under chilling stress in watermelon. Front Plant Sci.

[91] Ivanchenko MG, Zhu J, Wang B, Medvecká E, Du Y, Azzarello E, Mancuso S, Megraw M, Filichkin S, Dubrovsky JG (2015). The cyclophilin A DIAGEOTROPICA gene affects auxin transport in both root and shoot to control lateral root formation. Development.

[92] Mignolli F, Mariotti L, Picciarelli P, Vidoz ML (2017). Differential auxin transport and accumulation in the stem base lead to profuse adventitious root primordia formation in the aerial roots (aer) mutant of tomato (Solanum lycopersicum L.). J Plant Physiol.

[93] Keuskamp DH, Pollmann S, Voesenek LACJ, Peeters AJM, Pierik R (2010). Auxin transport through PIN-FORMED 3 (PIN3) controls shade avoidance and fitness during competition. Proc Natl Acad Sci.

[94] Peer WA, Hosein FN, Bandyopadhyay A, Makam SN, Otegui MS, Lee G-J, Blakeslee JJ, Cheng Y, Titapiwatanakun B, Yakubov B (2009). Mutation of the membrane-associated M1 protease APM1 results in distinct embryonic and seedling developmental defects in Arabidopsis. Plant Cell.

[95] Eliasson L (1978). Effects of nutrients and light on growth and root formation in Pisum sativum cuttings. Physiol Plant.

[96] Schenk ST, Schikora A (2015). Staining of callose depositions in root and leaf tissues. Bio-Protoc.

[97] Pan X, Welti R, Wang X (2010). Quantitative analysis of major plant hormones in crude plant extracts by high-performance liquid chromatography-mass spectrometry. Nat Protoc.

[98] Zhang J, Lin JE, Harris C, Pereira FCM, Wu F, Blakeslee JJ, Peer WA (2016). DAO1 catalyzes temporal and tissue-specific oxidative inactivation of auxin in Arabidopsis thaliana. Proc Natl Acad Sci.

[99] Ljvak KJ (2001). Analysis of relative gene expression data using real time quantitative PCR and the 2^<−ΔΔCT> method. Methods.

