# Supplementary material for: Auxin regulates adventitious root formation in tomato cuttings
Source: BMC Plant Biol. 2019 Oct 21;19:435. doi: 10.1186/s12870-019-2002-9 (PMC6802334; doi:10.1186/s12870-019-2002-9)
Supplement: Supplementary file 1 — Table S1. Reverse-phase high-performance liquid chromatography (HPLC) gradient parameters in mobile phases. (DOCX 13 kb) [file 12870_2019_2002_MOESM1_ESM.docx]

**Additional file 1: Table S1.** Reverse-phase high-performance liquid chromatography (HPLC) gradient parameters.

| **Time (min)** | **Gradient* (volume percentage)** |
| --- | --- |
| **0-2** | 30 |
| **2-20** | Increase linearly to 100 |
| **20-22** | 100 |
| **22-25** | Decrease linearly to 30 |

*****The binary solvent system uses water with 0.1% (vol/vol) formic acid and methanol with 0.1% (vol/vol) formic acid as mobile phases. Separations are performed by altering the percentage of mobile phases.
