# Supplementary material for: Auxin regulates adventitious root formation in tomato cuttings
Source: BMC Plant Biol. 2019 Oct 21;19:435. doi: 10.1186/s12870-019-2002-9 (PMC6802334; doi:10.1186/s12870-019-2002-9)
Supplement: Supplementary file 2 — Table S2. Conditions used for protonated or deprotonated plant hormones of Zeatin, ABA and SA ([M + H]+ or [M − H]−). (DOCX 14 kb) [file 12870_2019_2002_MOESM2_ESM.docx]

**Additional file 2: Table S2**. Conditions used for protonated or deprotonated plant hormones of Zeatin, ABA and SA ([M + H]^+^ or [M − H]^−^ ).

| **PH** | **SM** | **Q1** | **Q3** | **Q2(V)** | **RT*** |
| --- | --- | --- | --- | --- | --- |
| **Zeatin** | + | 220.2 | 136.2 | 29 | 2.91 |
| **ABA** | - | 262.8 | 152.6 | -22 | 4.29 |
| **SA** | - | 136.6 | 92.8 | -24 | 9.45 |

ABA, abscisic acid; SA, salicylic acid; Q1, precursor ion selected in Q1; Q3, product ion selected in Q3; Q2, collision energy (V); SM, scan mode.

*RT (retention time) listed in this table were obtained under HPLC and column conditions mentioned in the method.
