# Supplementary material for: Auxin regulates adventitious root formation in tomato cuttings
Source: BMC Plant Biol. 2019 Oct 21;19:435. doi: 10.1186/s12870-019-2002-9 (PMC6802334; doi:10.1186/s12870-019-2002-9)
Supplement: Supplementary file 3 — Table S3. Accession numbers of the genes used in this study. (DOCX 13 kb) [file 12870_2019_2002_MOESM3_ESM.docx]

**Additional file 3: Table S3**. Accession numbers of the genes used in this study.

| **Gene** | **NCBI Databases Accession** |
| --- | --- |
| *SlLAX1* | HQ671063.1 |
| *SlLAX2* | HQ671064.1 |
| *SlLAX3* | HQ671065.1 |
| *SlPIN1* | HQ127074.1 |
| *SlPIN2* | HQ127077.1 |
| *SlPIN3* | HQ127079.1 |
| *SlPIN4* | HQ127078.1 |
| *SlPIN5* | HQ127080.1 |
| *SlPIN6* | HQ127082.2 |
| *SlPIN7* | HQ127076.1 |
| *SlUBI3* | SX58253.1 |
