# Supplementary material for: Auxin regulates adventitious root formation in tomato cuttings
Source: BMC Plant Biol. 2019 Oct 21;19:435. doi: 10.1186/s12870-019-2002-9 (PMC6802334; doi:10.1186/s12870-019-2002-9)
Supplement: Supplementary file 4 — Table S4. Primers for qRT-PCR. (DOCX 14 kb) [file 12870_2019_2002_MOESM4_ESM.docx]

**Additional file 4 Table S4**. Primers for qRT-PCR.

| Gene | Primer 1 (5' - 3') | Primer 2 (5' - 3') |
| --- | --- | --- |
| *SlLAX1* | GTTGGACTGCTATGTATGTTATC | TGCTGGTGGAAGTGAAGG |
| *SlPIN2* | CGAGGAGGTAGGAGTATGAG | CACTTCCGCTTCCAACTTC |
| *SlPIN3* | GCTGCCGCTTCTATTATCG | CTCCTTAGCAAACACAAATGG |
| *SlPIN4* | AGTTATGGCTGCTGCTTC | CAAACACAAATGGGACAATC |
| *SlPIN7* | ATCAGCGGTCCAGCAGTC | GAACGATTCCTTGAGGTAGAGC |
| *SlUBI3* | TCAAGCACAAGAAGAAGAAGG | ACCGCACTCAGCATTAGG |
