# Supplementary material for: Auxin regulates adventitious root formation in tomato cuttings
Source: BMC Plant Biol. 2019 Oct 21;19:435. doi: 10.1186/s12870-019-2002-9 (PMC6802334; doi:10.1186/s12870-019-2002-9)
Supplement: Supplementary file 5 — Figure S1. The detailed elaboration of DR5pro:YFP localization in initiation phase of developing AR. (DOCX 301 kb) [file 12870_2019_2002_MOESM5_ESM.docx]

**
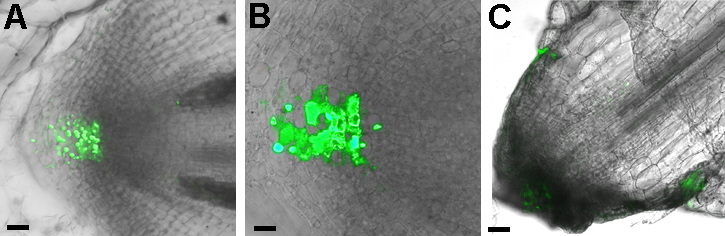
**

**Additional file 5: Figure S1.** DR5rp:YFP localization in initiation phase of developing AR and at emergence.

**(A)** DR5 localization in the developing AR. DR5:pro:YFP signals were mainly localized in the meristematic region of the AR primordium. **(B)** is 2X magnification of (A). (**C**) Emerged AR. Bar of A = 50 μm, B = 20 μm, C = 10 μm.
